# Physical activity, genetic predisposition and the risk of rheumatoid arthritis: a prospective cohort study

**DOI:** 10.3389/fspor.2026.1851796

**Published:** 2026-07-15

**Authors:** Yuanming Zhong, Song Lin, Chenyu Zhu, Xia Chen, Haomin Yang, Yan Wang, Jianhua Xu

**Affiliations:** 1 School of Physical Education and Sport Science, Fujian Normal University, Fuzhou, China; 2 Department of Clinical Nutrition, The Affiliated Huaian No.1 People’s Hospital of Nanjing Medical University, Huaian, China; 3 First Affiliated Hospital of Dalian Medical University, Dalian Medical University, Dalian, China; 4 Department of Epidemiology and Health Statistics, School of Public Health, Fujian Medical University, Fuzhou, China; 5 Fujian Key Laboratory of Developmental and Neural Biology, College of Life Sciences, Fujian Normal University, Fuzhou, China

**Keywords:** BMI - body mass index, CRP - C-reactive protein, physical activity, polygenic risk score (PGRS), rheumatoid arthritis

## Abstract

**Background:**

Physical activities have consistently been associated with rheumatoid arthritis risk. However, there is limited evidence regarding their associations according to different genetic susceptibility to the disease, and it remains poorly understood whether systemic inflammation and lipids may mediate this association.

**Methods:**

To investigate the association between physical activity and risk of rheumatoid arthritis, a multivariable Cox regression was performed using data from 351,857 participants in UK Biobank cohort. Additionally, we assessed the interaction of physical activity with polygenic risk score (PRS) for rheumatoid arthritis. We further estimated the percentage of total association between physical activity and rheumatoid arthritis that is mediated by C-reactive protein (CRP) and lipids.

**Results:**

A lower risk of rheumatoid arthritis was associated with the moderate and high level of physical activity (HR = 0.77, 95%CI = 0.70–0.84 and HR = 0.82, 95%CI = 0.75–0.89, respectively), and this association was stronger with five year after baseline. Separated by intensity of physical activity, only vigorous activity was associated with reduced risk of rheumatoid arthritis. A significant interaction was observed between physical activity and PRS for rheumatoid arthritis, indicating stronger association in those with low or medium genetic predisposition to rheumatoid arthritis. Furthermore, BMI and CRP was found to mediate 33.73% and 32.89% of the associations between physical activity and rheumatoid arthritis, while high-density lipoprotein (HDL) mediated 15.61% of the association.

**Conclusion:**

We observed an inverse association between physical activity and risk of rheumatoid arthritis in UK biobank, which was stronger in those with low or medium PRS. BMI, CRP and HDL may be key intermediates in the association, suggesting them as potential monitoring biomarker for physical activity intervention to prevent rheumatoid arthritis.

## 1 Introduction

Rheumatoid arthritis is an autoimmune musculoskeletal disorders with substantial burden on patients’ quality of life and public health systems (1). Given the rising incidence and disease burden associated with rheumatoid arthritis worldwide (2), identifying modifiable risk factors is critical for mitigating disease risk, such as physical activity, smoking, dietary habits, and body composition.

Population-based epidemiological studies suggests physical activity to be associated with rheumatoid arthritis risk (3–5), in which regular moderate to vigorous physical activity might have a protective effect against disease onset (6). Nevertheless, findings regarding the dose–response relationship and optimal intensity remain inconsistent across studies (3–5), potentially because of the difference in types of physical activities, follow-up duration, and heterogeneity in study populations (7). Furthermore, while the independent role of physical activity in autoimmune disease pathogenesis has been increasingly recognized (8, 9), limited research has explored its potential interaction with genetic factors, which may provide more precise and efficient preventive strategies.

Recent genome-wide association studies (GWAS) have identified numerous single-nucleotide polymorphisms (SNPs) associated with rheumatoid arthritis, enabling the construction of polygenic risk scores for individual risk stratification (10–12). However, it remains unclear whether the association with physical activity vary across strata of genetic predisposition to rheumatoid arthritis. Previous studies showed that increased physical activity can maintain a healthy weight and effectively reduce inflammation in the body (13–15), while recent studies have indicated that inflammation plays a pivotal role in the development of rheumatoid arthritis (16). Apart from inflammation, lipids may also change according to the status of physical activity (17, 18). However, to date, no studies have examined the extent to which physical activity influence the risk of rheumatoid arthritis through their impact on inflammation and lipid levels. A better understanding of the role of these markers may provide insights into the etiology of rheumatoid arthritis, and may support interventions aimed at modifiable factors to reduce the risk.

The aim of this study was to examine the association between physical activity and the risk of developing rheumatoid arthritis, and to explore whether this association differed according to genetic susceptibility, as quantified by a polygenic risk score for rheumatoid arthritis. We further assessed whether inflammation and lipid levels may mediate the relationship between physical activity and rheumatoid arthritis.

## 2 Methods

### 2.1 Study populations

**Figure 1:**
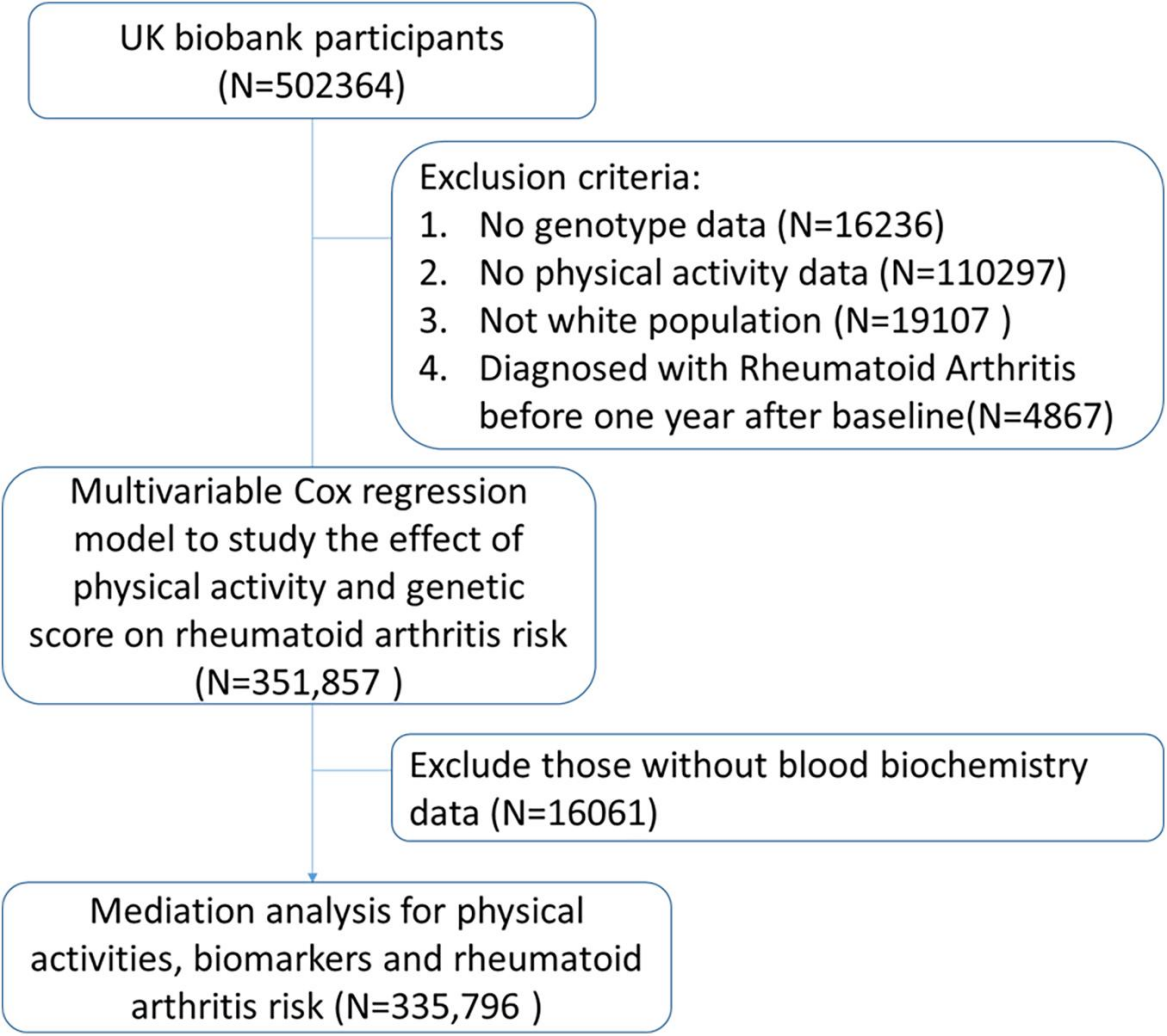
Flow chart for the study.

This research utilized data from the UK Biobank cohort, a prospective study enrolling over 500,000 individuals aged 40–69 years across 22 assessment centers throughout the United Kingdom from 2006 to 2010 (19). At baseline evaluation, extensive datasets covering sociodemographic characteristics, lifestyle factors, and health-related details were gathered via touchscreen questionnaires and face-to-face interviews. Additionally, comprehensive physical examinations were conducted, accompanied by the collection of biological specimens. The UKB was approved by The National Information Governance Board for Health and Social Care and the NHS North West Multicentre Research Ethics Committee (Ref: 11/NW/0382, 17 June 2011), and participants provided informed consent. This research was conducted using the UK Biobank Resource under Application 61083. The eligibility criteria defining the study population, including both inclusion and exclusion parameters, are illustrated in Figure 1. Those excluded participants were more likely to be older, with female sex, higher education level and Townsend deprivation index (Supplementary Table 1).

### 2.2 Physical activity

Physical activity levels were assessed using the International Physical Activity Questionnaire (IPAQ), which recorded the frequency and duration of walking, moderate-intensity exercise, and vigorous-intensity exercise undertaken in the previous week. Following the IPAQ scoring protocol (https://sites.google.com/site/theipaq/scoring-protocol), metabolic equivalent of task (MET) values of 8.0, 4.0, and 3.3 were allocated to vigorous activity, moderate activity, and walking, respectively (20). Weekly physical activity volume, expressed as MET-minutes per week, was computed by multiplying the duration and frequency of each activity by its corresponding MET value (21). In this study, physical activity was categorized as low, moderate and high according to the Guidelines for the data processing and analysis of the International Physical Activity Questionnaire. To further investigate the roles of different types of physical activities, we analyzed the walking, moderate-intensity exercise, and vigorous-intensity exercise MET-minutes per week separately and categorized them into tertiles.

### 2.3 Rheumatoid arthritis

Rheumatoid arthritis diagnosis was obtained by using unique personal identification numbers to link the cohort to the National Health Service (NHS) Digital for England and Wales, and National Records of Scotland, NHS Central Register for Scotland. The ICD-9 code 714, ICD-10 codes M05, M06 and M080 were used to identify rheumatoid arthritis diagnosis. The date of death was retrieved from death certificates held by the NHS Information Center and the NHS Central Register. Follow-up for the participants started from one year after date of enrollment (to avoid reverse causality issue) and continued until date of rheumatoid arthritis diagnosis, death, loss to follow-up or end of study (December 31, 2022), whichever occurred first. Participants with rheumatoid arthritis before the start of follow-up were excluded from the analysis.

### 2.4 Polygenic risk score

To assess whether genetic susceptibility interact with physical activity, we used the polygenic risk scores (PRS) calculated for rheumatoid arthritis on all individuals in UK Biobank. Details for the calculation of PRS can be found in the release note for the score (22). Briefly, for all individuals, a weighted PRS was calculated using the following formula:

$$PRS\;=\beta_{1}x_{1}\;+\beta_{2}x_{2}\;+\ldots .\beta_{k}x_{k}\;+\beta_{n}x_{n}$$

where *β* is the per-allele log odds ratio (OR) of rheumatoid arthritis-associated risk allele for SNP, x_k_ is the number of alleles for the same SNP (0, 1, 2), and *n* is the total number of the rheumatoid arthritis SNPs. For the analysis, we included only white participants of European ancestry, and the first five genetic principal components were incorporated into the models to adjust for potential confounding from population stratification.

### 2.5 Key covariates and mediating biomarkers

In UKB, a food frequency questionnaire was administered to assess participants’ dietary intake frequency and portion sizes during the previous 12 months. The validity and reproducibility of this 32-item food frequency questionnaire have been evaluated and verified (23). Key food groups included comprised fruits, vegetables, cereals, red meat (including pork, beef, and lamb), and processed meat, considering their potential associations with diseases. Each dietary component was assigned a score ranging from 0 to 0.5 points, yielding an overall healthy diet score between 0 and 2. Information on alcohol drinking frequency, adiposity measures (including body mass index, BMI), and smoking status was also obtained at baseline assessment.

Blood samples were collected from all participants at study enrollment. As part of the UK Biobank Biomarker Project, biomarker measurements were performed at the dedicated UK Biobank laboratory in Stockport. Comprehensive information regarding blood sample processing, storage, analytical procedures, and strict quality control protocols has been documented in previous publications (24, 25). In brief, serum levels of biochemical markers were quantified using immunoassay analyzers employing various techniques, including colorimetric, enzymatic rate, Chemiluminescent Immunoassay, and immune-turbidimetric assays. For the present analysis, C-reactive protein, total cholesterol, high-density lipoprotein (HDL), low-density lipoprotein (LDL), and triglycerides (TG) were selected as primary mediating biomarkers, most of which had a missing proportion below 10%. These biomarkers were treated as standardized continuous variables in the mediation analysis.

### 2.6 Statistical analysis

Multivariable Cox regression was used to investigate the association between physical activity and risk of rheumatoid arthritis with attained age as underlying time scale. This approach inherently adjusted for age-related risk throughout the follow-up period and accounted for the strong association of age with physical activity and rheumatoid arthritis risk (26).We further accounted for left truncation in data by including in the risk sets only persons at risk after their age at entry. Physical activity was categorized according to IPAQ guideline and the MET-minutes per week for walking, moderate-, and vigorous-intensity exercise were analyzed by tertiles and as standardized continuous variables. The effect of PRS for rheumatoid arthritis was also assessed by tertiles and as standardized continuous variables. We constructed two models: Model 1 adjusted for sex, assessment centers, education level, and Townsend deprivation index and Model 2 further adjusted for healthy diet score, sleep duration, alcohol consumption, smoking, and top 5 principal genetic components. To evaluate whether this association differed by genetic predisposition, we further performed stratified analyses according to the tertiles of PRS for rheumatoid arthritis. The proportional hazards assumption was tested using Schoenfeld residuals. To consider the potential influence from menopausal status, a sensitivity analysis was performed adding menopausal status into the model.

Further mediation analyses were performed using the med4way package (27) for BMI, CRP and the cholesterols. The overall excess risk can be divided into four parts, including the controlled direct effect, pure interaction, mediated interaction, and pure indirect effects. The formula for calculating the percentage of mediation is as follows: (*β*_mediated interaction_ + *β*_indirect effect_)/(*β*_direct effect_ + *βinteraction* + *β*_mediated interaction_ + *β*_indirect effect_). In the mediation model, linear regression was used for the association between physical activity and mediators, while Cox regression was used for the association between physical activity and rheumatoid arthritis. The analysis was adjusted for the sex, assessment centers, education level, Townsend deprivation index, healthy diet score, sleep duration, alcohol consumption and smoking. All analyses were two-sided and statistical analyses were performed using Stata 17.1.

## 3 Results

Over a median follow-up of 12.8 years, 3,864 of the 351,857 participants with complete data of physical activity and PRS developed rheumatoid arthritis, resulting to an incidence density of 0.88 per thousand person years. Participants with high physical activities were more likely to have high education level, less sleep duration and healthy diet (Table 1).

**Table 1:** Basic characteristics of the study population.

Characteristics	IPAQ group			
	Low	Medium	High	*P*-value
Age	56.44 ± 7.82	57.07 ± 8.02	56.91 ± 8.20	<0.001
Sex				<0.001
Male	31,601 (49.35)	65,282 (45.51)	72,707 (50.36)	
Female	32,439 (50.65)	78,159 (54.49)	71,669 (49.64)	
Education				<0.001
Lower qualification	22,902 (35.76)	55,238 (38.51)	46,852 (32.45)	
Middle qualification	16,355 (25.54)	35,706 (24.89)	33,973 (23.53)	
High qualification	24,551 (38.34)	52,023 (36.27)	62,917 (43.58)	
Missing	232 (0.36)	474 (0.33)	634 (0.44)	
Townsend deprivation index				<0.001
Lowest quartile	16,754 (26.16)	37,455 (26.11)	36,387 (25.2)	
Second quartile	16,371 (25.56)	36,739 (25.61)	37,015 (25.64)	
Third quartile	15,656 (24.45)	35,879 (25.01)	36,664 (25.39)	
Highest quartile	15,163 (23.68)	33,204 (23.15)	34,151 (23.65)	
Missing	96 (0.15)	164 (0.11)	159 (0.11)	
Smoking, *n* (%)				<0.001
Never	33,935 (52.99)	79,120 (55.16)	77,562 (53.72)	
Previous	22,226 (34.71)	50,377 (35.12)	52,627 (36.45)	
Current	7,624 (11.91)	13,483 (9.4)	13,676 (9.47)	
Missing	255 (0.4)	461 (0.32)	511 (0.35)	
Alcohol consumption, *n* (%)				<0.001
More than three times a week	28,251 (44.11)	69,290 (48.31)	68,114 (47.18)	
More than once a month	24,079 (37.6)	52,081 (36.31)	53,862 (37.31)	
Occasionally or never	11,586 (18.09)	21,867 (15.24)	22,189 (15.37)	
Missing	124 (0.19)	203 (0.14)	211 (0.15)	
Sleep duration (Hours)				<0.001
≤ 6	16,145 (25.21)	31,595 (22.03)	33,689 (23.33)	
7	24,650 (38.49)	58,521 (40.8)	57,255 (39.66)	
8	17,489 (27.31)	42,624 (29.72)	43,396 (30.06)	
≥ 9	5,559 (8.68)	10,471 (7.3)	9,831 (6.81)	
Missing	197 (0.31)	230 (0.16)	205 (0.14)	
Healthy Diet score				<0.001
0∼1	21,721 (33.92)	42,608 (29.7)	39,224 (27.17)	
1	29,201 (45.6)	64,261 (44.8)	59,626 (41.3)	
1∼2	13,038 (20.36)	36,468 (25.42)	45,390 (31.44)	
Missing	80 (0.12)	104 (0.07)	136 (0.09)	

In multivariable Cox regression analysis, a significant inverse association was observed between moderate or high level of physical activity and risk of rheumatoid arthritis (HR = 0.77, 95% CI = 0.70–0.84 for moderate activity group and HR = 0.82, 95% CI = 0.75–0.89 for high activity group). The association was stronger within 5 years after the enrollment, but attenuated whilst still significant more than 5 years after the baseline (Table 2).

**Table 2:** The associations between physical activity, genetic predisposition and risk of rheumatoid arthritis.

Variables			Model 1		Model 2	
	Total No.	No. of cases	HR (95% CI)	*P*-value	HR (95% CI)	*P*-value
IPAQ group						
Low	64,040	830	1.00 (REF)	<0.001	1.00 (REF)	<0.001
Moderate	1,43,441	1,449	0.74 (0.68–0.80)		0.77 (0.70–0.84)	
High	1,44,376	1,585	0.78 (0.71–0.85)		0.82 (0.75–0.89)	
By time since baseline						
< 5 years after baseline						
IPAQ group						
Low	64,040	207	1.00 (REF)	<0.001	1.00 (REF)	<0.001
Moderate	1,43,441	283	0.59 (0.49–0.70)		0.62 (0.52–0.75)	
High	1,44,376	296	0.60 (0.50–0.72)		0.64 (0.53–0.77)	
≥ 5 years after baseline						
IPAQ group						
Low	62,488	623	1.00 (REF)	0.013	1.00 (REF)	0.071
Moderate	1,40,945	1,166	0.79 (0.72–0.87)		0.82 (0.74–0.90)	
High	1,42,035	1,289	0.85 (0.77–0.93)		0.88 (0.80–0.97)	
By types of Activity						
MET for walking (min/week)						
Tertile 1	1,21,229	1,352	1.00 (REF)	0.410	1.00 (REF)	0.487
Tertile 2	1,17,764	1,192	0.86 (0.79–0.92)		0.87 (0.81–0.94)	
Tertile 3	1,12,864	1,320	0.93 (0.86–1.01)		0.94 (0.87–1.02)	
Std. continuous			1.01 (0.98–1.04)	0.520	1.01 (0.98–1.04)	0.510
MET for moderate activity (min/week)						
Tertile 1	1,34,653	1,415	1.00 (REF)	0.845	1.00 (REF)	0.567
Tertile 2	1,02,839	1,028	0.88 (0.82–0.96)		0.91 (0.84–0.98)	
Tertile 3	1,14,365	1,421	0.98 (0.91–1.05)		1.00 (0.92–1.07)	
Std. continuous			1.01 (0.98–1.04)	0.473	1.01 (0.98–1.04)	0.421
MET for vigorous activity (min/week)						
Tertile 1	1,37,456	1,851	1.00 (REF)	<0.001	1.00 (REF)	<0.001
Tertile 2	97,227	900	0.74 (0.69–0.80)		0.78 (0.72–0.85)	
Tertile 3	1,17,174	1,113	0.79 (0.73–0.85)		0.83 (0.77–0.89)	
Std. continuous			0.96 (0.93–0.99)	0.020	0.97 (0.94–1.00)	0.054
Genetic predisposition						
PRS for rheumatoid arthritis						
Tertile 1	1,17,286	991	1.00 (REF)	<0.001	1.00 (REF)	<0.001
Tertile 2	1,17,286	1,219	1.22 (1.12–1.33)		1.22 (1.12–1.32)	
Tertile 3	1,17,285	1,654	1.66 (1.53–1.79)		1.65 (1.53–1.79)	
Std. continuous			1.28 (1.24–1.32)	<0.001	1.28 (1.24–1.32)	<0.001

Model 1 adjusted for sex, assessment centers, education level, and Townsend deprivation index and Model 2 further adjusted for healthy diet score, sleep duration, alcohol consumption, smoking, and top 5 principal genetic components.

When stratified by types of activities, METs for walking or moderate activity did not show significant association with risk of rheumatoid arthritis overall. Only participants in the second tertile were associated with decreased rheumatoid arthritis risk. On the contrary, participants with tertile 2 and tertile 3 MET for vigorous activity had a lower risk of developing rheumatoid arthritis, although the association attenuated slightly in the third tertile (HR = 0.78, 95% CI = 0.72–0.85 for Tertile 2 and HR = 0.83, 95% CI = 0.77–0.89 for Tertile 3). Trend test suggested a clear linear trend for the association between vigorous activity and rheumatoid arthritis risk (*p* < 0.001), while the result for standardized continuous MET is borderline significant. Sensitivity analysis adding menopausal status into the model did not change the results (Supplementary Table 2).

When investigating the association between PRS and rheumatoid arthritis, a clear significant association was observed both for the trend test and standardized continuous PRS (HR = 1.28, 95% CI = 1.24–1.32, *p* < 0.001).

**Figure 2:**
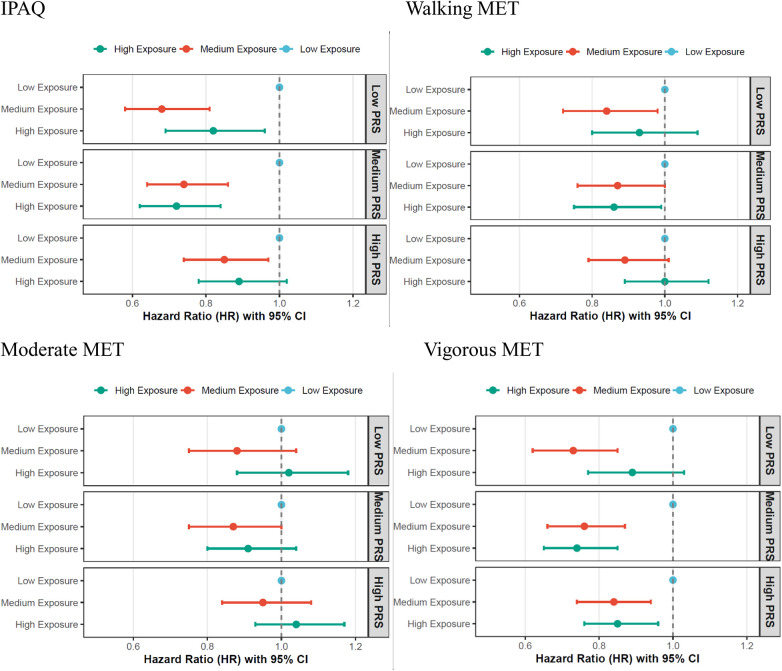
Stratified analysis and the interaction of physical activity, genetic predisposition and risk of rheumatoid arthritis.

In the stratified analysis by tertiles of rheumatoid arthritis PRS, we found an interaction between tertiles of PRS and IPAQ groups (*p* for interaction = 0.049, Figure 2, Supplementary Table 3), suggesting a stronger inverse association of physical activity among the low and medium PRS groups (strongest HR = 0.68, 95% CI = 0.58–0.81). However, in the high PRS group, even participants with high physical activity did not have significantly reduced risk of rheumatoid arthritis (HR = 0.89, 95% CI = 0.78–1.02). For the specific types of physical activities, there is no clear interaction identified. However, a stronger association of vigorous MET was still observed among participants with low or medium PRS.

**Figure 3:**
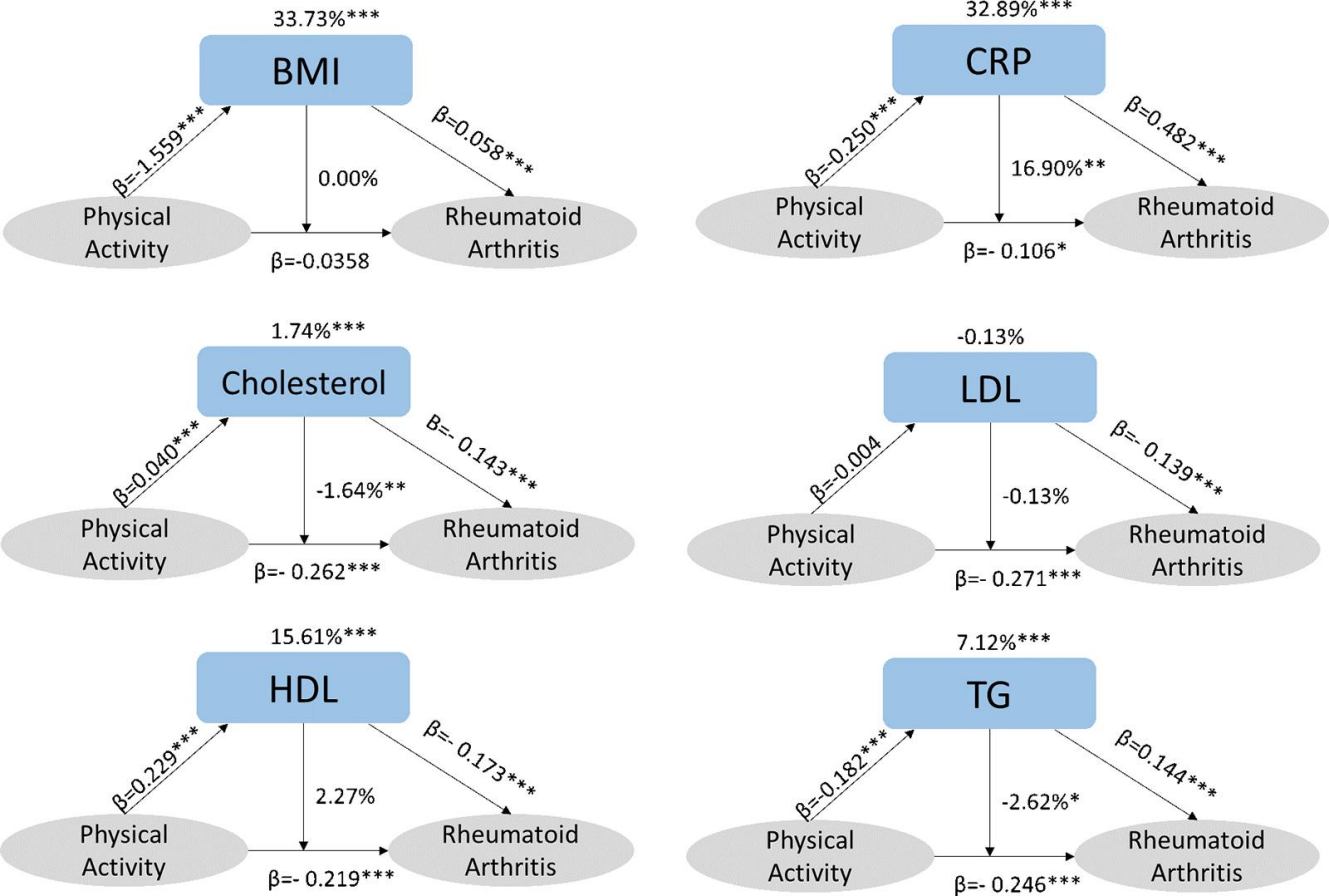
Mediation biomarkers for the association between physical activity and rheumatoid arthritis. * means *P* < 0.05, ** means *P* < 0.01 and *** means *P* < 0.001.

When considering the mediation analysis, we found a strong association of physical activity with BMI, CRP and total cholesterol. BMI mediated 33.73% of the association between physical activity and risk of rheumatoid arthritis, while CRP mediated 32.89% of the association (*p* for mediators < 0.001, Figure 3). For cholesterols, total cholesterol mediated only 1.74% of the association and the significant mediator was HDL (15.61%).

## 4 Discussion

Moderate or high level of physical activity may reduce rheumatoid arthritis risk, with stronger association within 5 years. Among different types of activities, only vigorous activity showed the inverse association. Higher PRS increased RA risk, and the association with physical activity was only observed among the participants with low/medium PRS. BMI and CRP each mediated about one-third of the protective association, while HDL mediated 15.61%.

In UK Biobank, we found that participants with moderate or high level of physical activity had lower risk of rheumatoid arthritis. Similarly, several observational studies have found that physical activity is associated with risk of rheumatoid arthritis and other autoimmune diseases in the nervous and musculoskeletal systems (3–5, 28, 29), and the association was confirmed by a meta-analysis (7). Our result showed stronger association during the short term, while longer time association was still observed after five years. Although reverse causality might influence the estimation for the short-term association between physical activity and rheumatoid arthritis risk, our findings suggested a consistent long-term association of physical activity with rheumatoid arthritis. In addition, the attenuated estimates might also be explained by the potential changing of physical activity after baseline.

When stratified by types of physical activities, only participants in the second tertile of METs for walking or moderate activity were inversely associated with rheumatoid arthritis risk. This might be explained by the fact that excessive moderate activity in the highest tertile may induce mechanical joint overload and transient inflammatory stress, offsetting the benefits of exercise and abolishing the protective association. The observed association between vigorous physical activities and rheumatoid arthritis aligns with previous findings indicating moderate to vigorous physical activities may reduce risk of rheumatoid arthritis (4, 5), while the attenuated association in the third tertile may also be explained by the mechanical joint overload and transient inflammatory stress similarly. Vigorous physical activities may reduce inflammation in individuals and therefore reduce the risk of rheumatoid arthritis (30). However, only vigorous activity consistently improves insulin sensitivity, reduces visceral fat, and lowers chronic low-grade inflammation, while moderate activity often does not reach the threshold needed to alter (31). This may also explain why we did not observe the association with moderate activities.

We confirmed the genetic association between PRS and rheumatoid arthritis in UK biobank. This is not surprising considering the more than 50% heritability of rheumatoid arthritis (12). Interestingly, we also observed borderline significant interaction between PRS and physical activity, showing a slightly stronger inverse association in those with lower genetic predisposition to rheumatoid arthritis, which has not been reported previously. As rheumatoid arthritis is a complex disease mediated by multiple risk factors (32), individuals with a stronger genetic predisposition to rheumatoid arthritis might be less likely to modify their disease risk through lifestyle interventions. However, a 10%–15% reduced risk was observed within this group of population, suggesting that physical activity may still be part of the intervention package.

In the present study, we uncovered novel mediating pathways for the association between physical activity and rheumatoid arthritis through BMI and CRP, each accounting for one-third of the association. BMI is a known risk factor for rheumatoid arthritis (33) and it's plausible that moderate to vigorous physical activity may decrease BMI and therefore reduce risk of rheumatoid arthritis. Notably, CRP also mediated one third of the association, suggesting that inflammation might be a possible mechanism for physical activity related risk of rheumatoid arthritis. CRP is synthesized in the liver under the stimulation of pro-inflammatory cytokines such as IL-6, IL-1β, and TNF-α. As a biomarker of systemic inflammation, it is directly involved in joint damage, vascular complications, and disease progression in rheumatoid arthritis (34, 35). Physical activity may reduce visceral adiposity, which serves as the primary source of chronic IL-6 (36), thereby interrupting the upstream driver of CRP production, and reduce risk of rheumatoid arthritis.

The mediation of cholesterols might be also related to their inflammatory effect. Total cholesterol promotes joint inflammation in rheumatoid arthritis by enhancing pro-inflammatory signaling, inducing oxidative stress, and activating autoimmune responses (37). In contrast, HDL exerts anti-inflammatory, antioxidant, and immunomodulatory effects (38). HDL can inhibit the activation of autoreactive T cells and promote the polarization of anti-inflammatory macrophages (39), thereby playing an important role in maintaining immune homeostasis and preventing autoimmune injuries. However, all these biomarkers, including CRP and BMI were measured at baseline together with physical activity, and temporality of them cannot be ascertained. Therefore, the potential mediated association by these biomarkers should be interpreted with caution.

To our knowledge, this is the largest population-based cohort study to date to investigate the potential association between different types of physical activity and rheumatoid arthritis risk. Other strengths included that UKB provided adequate information on blood biomarker to investigate the mediators. Our study is also subject to several limitations. Diagnosis of rheumatoid arthritis relied on ICD codes from hospitalization records, which resulted in a positive predictive value of about 79% (40). While this method may overestimate the absolute disease risk, it would not substantially bias the observed associations. In addition, limited by the ICD codes, we were unable to determine the specific joint sites affected by rheumatoid arthritis. Biomarkers such as CRP may capture both acute and chronic inflammation. Given that acute inflammatory status varies over time, it may cause potential misclassification. We presumed this misclassification to be random, which could have attenuated the estimated associations. This study only included European white population and the excluded participants were more likely to be female with higher education level and Townsend deprivation index, which might impair the generalizability of our results. Therefore, caution is warranted when extrapolating our conclusions to non-Caucasian ethnic groups, and further study should be performed to validate our findings in other conditions.

## 5 Conclusion

In conclusion, we found that physical activity might reduce the risk of rheumatoid arthritis, especially for vigorous activities. The association was stronger for the short term, while attenuated in the long term for more than five years. PRS was associated with increased risk of rheumatoid arthritis, and interact with physical activity, resulting stronger association of physical activity among those with low or moderate genetic score. In addition to BMI, CRP and HDL also mediated the association between physical activity and rheumatoid arthritis risk, suggesting them as key biomarkers to monitoring the role of physical activity in preventing rheumatoid arthritis.

## Data Availability

The data analyzed in this study is subject to the following licenses/restrictions: Data from UK Biobank are available to researchers upon application. Requests to access these datasets should be directed to https://www.ukbiobank.ac.uk/.
