# Supplementary material for: Physical activity, genetic predisposition and the risk of rheumatoid arthritis: a prospective cohort study
Source: Front Sports Act Living. 2026 Jul 15;8:1851796. doi: 10.3389/fspor.2026.1851796 (PMC13416464; doi:10.3389/fspor.2026.1851796)
Supplement: Supplementary file 1 [file Table1.docx]

**Supplementary Table 1.Basic characteristics of the included and excluded participants**

|  | Excluded  (N=142,846) | Included  (N=351,857) | P-value |
| --- | --- | --- | --- |
| **Age** | 57.23±8.17 | 56.89±8.06 | <0.001 |
| **Sex** |  |  | <0.001 |
| Male | 56734 (39.72) | 169590 (48.2) |  |
| Female | 86112 (60.28) | 182267 (51.8) |  |
| **Education** |  |  | <0.001 |
| Lower qualification | 34474 (24.13) | 124992 (35.52) |  |
| Middle qualification | 33798 (23.66) | 86034 (24.45) |  |
| High qualification | 71426 (50.00) | 139491 (39.64) |  |
| Missing | 3148 (2.2) | 1340 (0.38) |  |
| **Townsend deprivation index** |  |  | <0.001 |
| Lowest quartile | 29248 (20.48) | 94288 (26.8) |  |
| Second quartile | 31560 (22.09) | 91955 (26.13) |  |
| Third quartile | 35312 (24.72) | 88213 (25.07) |  |
| Highest quartile | 46535 (32.58) | 76982 (21.88) |  |
| Missing | 191 (0.13) | 419 (0.12) |  |
| **Smoking, n (%)** |  |  | <0.001 |
| Never | 79075 (55.36) | 190617 (54.17) |  |
| Previous | 44472 (31.13) | 125230 (35.59) |  |
| Current | 17072 (11.95) | 34783 (9.89) |  |
| Missing | 2227 (1.56) | 1227 (0.35) |  |
| **Alcohol consumption, n (%)** |  |  | <0.001 |
| More than three times a week | 48827 (34.18) | 165655 (47.08) |  |
| More than once a month | 52119 (36.49) | 130022 (36.95) |  |
| Occasionally or never | 40378 (28.27) | 55642 (15.81) |  |
| Missing | 1522 (1.07) | 538 (0.15) |  |
| **Sleep duration (Hours)** |  |  | <0.001 |
| ≤6 | 39576 (27.71) | 81429 (23.14) |  |
| 7 | 49582 (34.71) | 140426 (39.91) |  |
| 8 | 38730 (27.11) | 103509 (29.42) |  |
| ≥9 | 11515 (8.06) | 25861 (7.35) |  |
| Missing | 3443 (2.41) | 632 (0.18) |  |
| **Healthy Diet score** |  |  | <0.001 |
| 0~1 | 42766 (29.94) | 103553 (29.43) |  |
| 1 | 60861 (42.61) | 153088 (43.51) |  |
| 1~2 | 36681 (25.68) | 94896 (26.97) |  |
| Missing | 2538 (1.78) | 320 (0.09) |  |

**Supplementary Table 2.The associations between physical activity and risk of rheumatoid arthritis, with additional adjustment for menopausal status**

|  |  |  | Model 2 | |  | Adjusting for menopausal status | |
| --- | --- | --- | --- | --- | --- | --- | --- |
|  | Total No. | No. of cases | HR (95% CI) | P-value |  | HR (95% CI) | P-value |
| IPAQ group |  |  |  |  |  |  |  |
| Low | 64040 | 830 | 1.00 (REF) | <0.001 |  | 1.00 (REF) | <0.001 |
| Moderate | 143441 | 1449 | 0.77 (0.70-0.84) |  |  | 0.77 (0.71-0.84) |  |
| High | 144376 | 1585 | 0.82 (0.75-0.89) |  |  | 0.82 (0.75-0.89) |  |
| By types of Activity |  |  |  |  |  |  |  |
| MET for walking (min/week) |  |  |  |  |  |  |  |
| Tertile 1 | 121229 | 1352 | 1.00 (REF) | 0.487 |  | 1.00 (REF) | 0.532 |
| Tertile 2 | 117764 | 1192 | 0.87 (0.81-0.94) |  |  | 0.87 (0.81-0.94) |  |
| Tertile 3 | 112864 | 1320 | 0.94 (0.87-1.02) |  |  | 0.94 (0.87-1.02) |  |
| Std. continuous |  |  | 1.01 (0.98-1.04) | 0.510 |  | 1.01 (0.98-1.04) | 0.471 |
| MET for moderate activity (min/week) |  |  |  |  |  |  |  |
| Tertile 1 | 134653 | 1415 | 1.00 (REF) | 0.567 |  | 1.00 (REF) | 0.481 |
| Tertile 2 | 102839 | 1028 | 0.91 (0.84-0.98) |  |  | 0.91 (0.84-0.99) |  |
| Tertile 3 | 114365 | 1421 | 1.00 (0.92-1.07) |  |  | 1.00 (0.93-1.08) |  |
| Std. continuous |  |  | 1.01 (0.98-1.04) | 0.421 |  | 1.01 (0.98-1.04) | 0.365 |
| MET for vigorous activity (min/week) |  |  |  |  |  |  |  |
| Tertile 1 | 137456 | 1851 | 1.00 (REF) | <0.001 |  | 1.00 (REF) | <0.001 |
| Tertile 2 | 97227 | 900 | 0.78 (0.72-0.85) |  |  | 0.79 (0.72-0.85) |  |
| Tertile 3 | 117174 | 1113 | 0.83 (0.77-0.89) |  |  | 0.83 (0.77-0.90) |  |
| Std. continuous |  |  | 0.97 (0.94-1.00) | 0.054 |  | 0.97 (0.94-1.00) | 0.062 |

Supplementary Table 3 The stratified analysis and the interaction of physical activity, genetic predisposition and risk of rheumatoid arthritis

|  | Low PRS group |  | Medium PRS group | | High PRS group | P for interaction |
| --- | --- | --- | --- | --- | --- | --- |
|  | HR (95%CI) |  | HR (95%CI) | | HR (95%CI) |  |
| IPAQ groups |  |  |  |  |  |  |
| Low |  |  |  |  |  | 0.049 |
| Medium | 0.68 (0.58-0.81) | | 0.74 (0.64-0.86) | | 0.85 (0.74-0.97) |  |
| High | 0.82 (0.69-0.96) | | 0.72 (0.62-0.84) | | 0.89 (0.78-1.02) |  |
| Walking MET |  |  |  |  |  | 0.957 |
| Low |  |  |  |  |  |  |
| Medium | 0.84 (0.72-0.98) | | 0.87 (0.76-1.00) | | 0.89 (0.79-1.01) |  |
| High | 0.93 (0.8-1.09) | | 0.86 (0.75-0.99) | | 1.00 (0.89-1.12) |  |
| Moderate MET |  |  |  |  |  | 0.718 |
| Low |  |  |  |  |  |  |
| Medium | 0.88 (0.75-1.04) | | 0.87 (0.75-1.00) | | 0.95 (0.84-1.08) |  |
| High | 1.02 (0.88-1.18) | | 0.91 (0.80-1.04) | | 1.04 (0.93-1.17) |  |
| Vigorous MET |  |  |  |  |  |  |
| Low |  |  |  |  |  | 0.839 |
| Medium | 0.73 (0.62-0.85) | | 0.76 (0.66-0.87) | | 0.84 (0.74-0.94) |  |
| High | 0.89 (0.77-1.03) | | 0.74 (0.65-0.85) | | 0.85 (0.76-0.96) |  |
